# A three-year randomized clinical trial evaluating direct posterior composite restorations placed with three self-etch adhesives

**DOI:** 10.1080/26415275.2021.1939034

**Published:** 2021-06-25

**Authors:** Joseph Sabbagh, Layal El Masri, Jean Claude Fahd, Paul Nahas

**Affiliations:** Department of Restorative and aesthetic dentistry and Endodontics, Faculty of Dental Medicine, Lebanese University, Beirut, Lebanon

**Keywords:** Composite restorations, *in vivo* study, posterior teeth, self-etch adhesives, universal adhesives, USPHS criteria

## Abstract

**Aim:**

To compare the clinical performance of composite restorations placed with a universal adhesive, one-step and two-step self-etch adhesives in class I and II posterior cavities.

**Materials and methods:**

In this *in vivo* study, 46 volunteers presenting with at least three carious lesions were included. Each participant received the three restorative systems: universal adhesive/nanofilled composite (Scotchbond Universal/Filtek Z350 XT: SBU/FZXT), one-step self-etch adhesive/microhybrid composite (G-aenial bond/G-aenial Posterior: GB/GP) and the two-step self-etch adhesive/nanohybrid composite (OptiBond XTR/Herculite Ultra: OBX/HU). The adhesives were all placed in self-etch mode. In total, 138 restorations were evaluated at baseline and at 6,12 and 36 months using the modified United States Public Health Service criteria. Data were analysed using Kruskal–Wallis, Mann–Whitney U, Friedman and Wilcoxon non-parametric tests (*p* < .05). Ninety-one restorations were evaluated at 36 months.

**Results:**

Seven restorations, three SBU/FZXT, three GB/GP and one OBX/HU failed during this study. The reasons for failure were marginal fracture and secondary caries. SBU/FZXT restorations showed significant marginal deterioration in all parameters. Overall success rates were: 93.5% (SBU/FZXT), 96.6% (GB/GP) and 96.8% (OBX/HU).

**Conclusions:**

After three years, the three restorative systems have comparable clinical effectiveness and success rates, except for the marginal integrity, that was suboptimal for both the SBU/FZXT and GB/GP restorations in comparison to the OBX/HU restorations.

## Introduction

Resin composites and adhesives are considered state of art in today’s restorative dentistry. These materials have evolved rapidly since they were first introduced, resulting in more durable and aesthetic restorations [1]. This led to a paradigm shift in dentistry, away from amalgam towards the more tooth-structure preserving composites [2]. However, there are various setbacks that are composite-related, including wear, microleakage, discoloration, postoperative sensitivity and polymerization shrinkage [3,4]. In an attempt to overcome the aforementioned problems, different formulations of resin composites were developed with the aid of nanotechnology such as nanofilled and nanohybrid composites. Manufacturers claimed that these composites have improved physical properties, superior polishability and better gloss retention, than the commonly used microhybrid resin composites. However, recent studies, including systematic reviews and meta-analyses, have not corroborated these claims [5,6]. Moreover, similar clinical behaviour was observed for nanocomposites and microhybrid composites [2].

In parallel to the development of resin composites, the adhesive systems have significantly evolved, simplifying application procedures, hence more user-friendly. In addition, self-etch (SE) adhesives were sought improved through changes in their photo-initiators and by the addition of functional monomers. Although, they imply a shorter application protocol, but their ability to adequately etch enamel has been questioned [7]. Strong SE adhesives were then introduced, which bonded effectively to enamel but formed weak dentin bonds and have been associated with a higher annual failure rate (5.4%) when compared to mild SE adhesives (3.6%) [8]. As a consequence, the idea of selectively etching the enamel with phosphoric acid prior to the application of mild SE adhesives was born [7].

A recent development in adhesive systems is the universal adhesives (UAs). They are one-step SE adhesives, which can be applied using an etch-and-rinse, self-etch or selective enamel-etch mode [7]. This allows dentists to select their adhesive technique according to their preference and the clinical situation [9,10]. Universal adhesives range from ultra-mild (pH ≥ 2.5) to mild (pH ≈ 2) and contain functional phosphate and/or carboxylate monomers that can bond chemically to dental tissues [7,11–13]. According to *in vitro* studies, their bond strength to dentin is determined by the pH, while the bond to enamel is enhanced when used in selective enamel-etch mode [14]. This was confirmed by a five-year clinical assessment, in which the universal adhesive performance was superior when applied using the etch and rinse (E&R) mode compared to the SE mode [15]. Existing data, have shown that the two-step SE adhesives, form a more stable bond and durable restorations than the simplified SE adhesives [8]. Simplified adhesives are well accepted by dentists and are available from numerous manufacturers. But presently, there is no agreement in the literature concerning the best adhesive for clinical use in the restoration of Class I and II posterior cavities.

The purpose of this randomized clinical trial was to compare the performance of nanofilled, microhybrid and nanohybrid resin composites bonded respectively with a universal adhesive, a one-step and a two-step self-etch adhesives in Class I and II posterior cavities, after thirty-six months, using the modified United Stated Public Health Services criteria (USPHS). The null hypothesis (H0) tested was that there would be no differences in the clinical performance for the three restorative systems after 3 years.

## Materials and methods

### Study design

**Figure 1.**
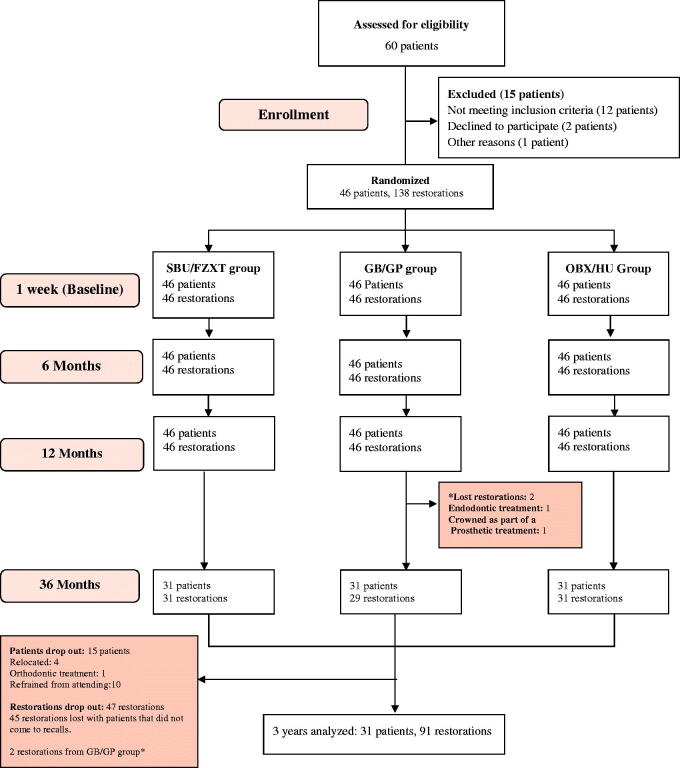
CONSORT Flow diagram.

This randomized trial was a single-site study conducted at university dental clinics in the Department of Restorative and Aesthetic Dentistry, Faculty of Dental Medicine, Lebanese University, Lebanon. The study was designed according to the Consolidated Standards of Reporting Trials (CONSORT) statement (Figure 1). A consent form and the protocol were submitted, reviewed and approved by the Ethical Committee of the Lebanese University. All procedures were performed according to the ethical standards of the institutional and/or national research committee and world medical association Declaration of Helsinki [16]. Selected patients were assessed for eligibility for participation, using the following inclusion criteria: (1) teeth with shallow to moderate Class I or II carious lesions, (2) a good periodontal status, (3) the absence of pulp pathology, (4) the absence of restorations on selected teeth, (5) the absence of parafunctional habits, (6) the included teeth were vital. Patients were healthy, physically and mentally with a non-compromised medical history and with the absence of allergic history to methacrylate. Patients that did not fit any of these criteria were excluded from the study. They were informed with the clinical procedure and signed an informed consent form.

### Restorative procedure

This clinical study included 138 posterior teeth in 46 patients (22 males and 24 females) with three cavities in each patient. The age range of the patients was between 20–55 years. The three cavities were randomly restored using the three different restorative systems. For each cavity in each patient, a coin was tossed to randomize the selection of materials. Heads denoted the Universal one-step self-etch adhesive and a nanofilled composite (SBU/FZXT); tails denoted the one-step self-etch adhesive and a microfilled composite (GB/GP); and the third cavity was assigned to the two-step self-etch adhesive and a nanohybrid composite (OBX/HBU). A modification to the randomization process within patients was made to ensure the equal distribution of the materials among tooth type and class type (Tables 1 and 2). Operators were not blinded to the materials used, as they can be easily identified from the application protocol. The sample size of this study (46 teeth per material) was chosen according to the recommendations of the ADA Acceptance Guidelines, where at least 30 restorations per material is required in the original study design [17].

**Table 1.** Distribution of cavity class among groups.

Cavity Class	Restorative systems			Sig.
	SBU/FZXT	GB/GP	OBX/HU	
Class I (with Buccal, palatal or lingual extension)	3 (6.5%)	3 (6.5%)	1 (2.2%)	*0.871*Fisher Exact Test
Class I	11 (23.9%)	12 (26.1%)	12 (26.1%)	
Class II OD, OM	21 (45.7%)	20 (43.5%)	20 (43.5%)	
Class II MOD	11 (23.9%)	11 (23.9%)	13 (28.3%)	
Total	46 (100.0%)	46 (100.0%)	46 (100.0%)	

**Table 2.** Distribution of tooth types among groups.

	Restorative systems			Sig.
Tooth	SBU/FZXT	GB/GP	OBX/HU	
Premolar	21 (45.2%)	19 (45.2%)	20 (51.6%)	*0.971*Fisher Exact Test
Molar	25 (54.8%)	27 (54.8%)	26 (48.4%)	
Total	46 (100.0%)	46 (100.0%)	46 (100.0%)	

Three calibrated dental residents from the Department of Restorative and Aesthetic Dentistry Department, performed the operative procedures. Bite-wing radiographs were taken before cavity preparation and the resin composite shade was selected. Local anaesthesia (Septanest,1:200.000, Septodont, Saint-Maur-des - Fossés, France) was given to patients as needed to avoid discomfort during the restorative procedure. Conservative cavities were prepared, using cylindrical diamond burs (SS White, USA) at high-speed with water cooling and round carbide burs (SS White, USA) at low speed were used to remove caries. The cavity preparations were shallow to moderate therefore a liner or base was not indicated. The location of the cervical margins was not recorded. Rubber dam was used to isolate the operative field, to provide ideal clinical conditions. For class II restorations, a pre-mounted metallic matrix system (SuperMat, Kavo-Kerr, Bioggio, Switzerland) and wooden wedges were placed to restore the anatomical shape and the proximal contact of the teeth. The restorations were distributed within one patient between carious molars and premolars (3 molars or 3 premolars or 1 molar and 2 premolars or 2 premolars and 1 molar). Cavities were randomly distributed to the following test restorative systems: SBU/FZXT: 1-step self-etch universal adhesive, Scotch Bond universal and a nanofilled composite, Filtek Z350 XT (3 M ESPE; Seefeld, Germany), GB/GP: 1-step self-etch adhesive, G-aenial bond and a microfilled hybrid composite, G-aenial Posterior (GC Corporation; Tokyo, Japan), OBX/HU: 2-step self-etch adhesive, OptiBond XTR and a nanohybrid composite, Herculite Ultra (Kavo Kerr Corp; Orange, CA, USA), (Table 3). The adhesives were all used in a self-etch mode. The three restorative systems (adhesive/composite) were placed in every patient to provide same subject control. Following the application of the adhesive systems as per manufacturer instructions, the resin composites were placed by the layering technique (2-mm-thick increments) and light-cured for 20 s using a Demi LED light curing device (Demetron, Kerr, USA). Finishing and polishing procedure was performed using carbide finishing burs and Identoflex and Occlubrush points (Kavo- Kerr, Bioggio, Switzerland).

**Table 3.** Name, composition, application and batch numbers of used materials.

Adhesive	Composition and pH^a^	Application^a^	Resin Composite	Composite Composition^a^
**Scotchbond Universal** (3M ESPE, Seefeld, Germany) One-bottle system self- etch *Lot 532117*	**Adhesive** MDP Phosphate monomer, Dimethacrylate Resins, HEMA,Vitrebond copolymer, Filler, Ethanol, Water, Intiators, Silane. *Ultra-mild* (pH≈2.7)	1.Apply scotch bond universal adhesive to prepared tooth. 2. Rub for 20 seconds. 3.Gently air-dry adhesive for 5 seconds to evaporate solvent. 4. Light cure for 10 seconds.	**Filtek Z350** *Lot 497440*	**Resin** UDMA, Bis-EMA, Bis-GMA, TEGDMA **Fillers** Silica (20 nm non-agglomerated/aggregated) zirconia (4–11 nm non-agglomerated/aggregated and agglomerated) clusters, zirconia/ silica aggregated particles (20 nm silica particles combined with 4-11 nm zirconia.
**G-aenial Bond** (GC Corporation, Tokyo, JAPAN) One-bottle system self- etch *Lot 1410101*	4-META Phosphoric acid ester monomer, Dimethacrylate monomers, Distilled water, Acetone, Silicon dioxide, Photo-initiator *Intermediately strong* (pH≈1.5)	1.Shake bottle of G-Bond 2. Apply to the enamel and dentin using disposable applicator brushes. 3. Leave undisturbed for 10 seconds after application. 4. Dry thoroughly for 5 Seconds 5.Light cure for 10 seconds.	**G-aenial posterior** *Lot 201204091*	**Resin** UDMA Dimethacrylate co-monomers **Fillers** milled pre-polymerized fillers averaging 16 to 17μ in size. Fluoroaluminosilicate glass. Fumed silica is dispersed between the pre-polymerized fillers and the other inorganic fillers.
**OptiBond XTR** (Kavo Kerr; Orange, CA, USA) Two-bottle system Self-etch *Lot 4866387 Lot 4866388*	**Primer:** Glycerol diphosphate monomer (GPDM), hydrophilic co-monomers and di-functional methacrylate monomers, water, acetone, ethyl alcohol, camphorquinone (pH≈2.4) **Adhesive:** hydrophobic cross-linking monomers, ethyl alcohol, camphorquinone, 0.4-μm barium glass 15%, sodium hexafluorosilicate *Mild*(pH≈3.4)	1. Apply OptiBond XTR Primer to enamel/dentin using scrubbing motion. 2. Air thin with medium pressure. 3. Shake OptiBond XTR adhesive briefly. Apply to enamel/dentin surface using light brushing motion. 4.Air thin with medium air pressure and then strong air for at least 5 seconds. 5. Light cure for 10 seconds. 6. Place composite according to manufacturer’s instructions for use and light cure.	**Herculite Ultra** *Lot 3318302*	**Resin** Uncured Methacrylate Ester Monomers **Fillers** Three fillers—prepolymerized filler (PPF), silica nanofillers (20 − 50 nm), Barium glass (0.4 micron).

HEMA: Hydroxyethylmethacrylate; MDP: Methacryloyloxydecyl dihydrogen phosphate; TEGDMA: trietyleneglycoldimethacrylate; UDMA: Urethanedimethacrylate; GPDM: Glycerol-phosphate-dimethacrylate; Bis-GMA: Bisphenol A-glycidyl methacrylate; Bis-EMA: Bisphenol A ethoxylate dimethacrylates; 4-META: 4-methacryloxyethyl trimellitate anhydride.

^a^According to manufacturers’ MSDS (material safety data sheet).

### Evaluation

Restorations were evaluated at baseline and at six, 12 and 36 months, by two calibrated examiners, that were blinded to the tested groups using the modified USPHS criteria (Table 4) [18]. The evaluations included mirrors, probes, radiographs and intra oral photographs. When a difference in scores was observed between evaluators, a third assessor evaluated the restorations and a consensus was reached.

**Table 4.** Modified US Public Health Service criteria for direct clinical evaluation of restorations.

Criteria	Definition
Anatomical form	A: Restoration is continuous with existing anatomical form. B: Restoration is discontinuous with existing anatomical form but missing material is not sufficient to expose dentin or base. C: sufficient material is lost to expose dentin or base.
Marginal adaptation	A: The explorer does not catch. B: Explorer catches, no crevice is visible in to which explorer will penetrate. C: Obvious crevice at margin, enamel, dentin or base exposed. D: Restoration is mobile, fractured or missing.
Color match	A: Restoration matches the shade and translucency of adjacent tooth structure. B: Perceptible mismatch, but mismatch is within the normal range of tooth shades. C: The mismatch is outside the normal range of tooth shades and translucency.
Marginal discoloration	A: No discoloration anywhere along the margin. B: Superficial staining (removable, usually localized). C: Deep staining (not removable, generalized).
Surface texture	A: No surface roughness or pits. B: Slight surface roughness or pitted surface. C: Obvious surface roughness or pitted surface.
Secondary caries	A: No caries is present. D: Caries is present.

*A: Alpha; B: Bravo; C: Charlie; D: Delta*.

### Statistical analysis

Data were assessed using descriptive statistics. The Kruskal–Wallis and Mann–Whitney U non-parametric tests were used to compare the performance of the three restorative systems at each evaluation time (baseline, 6 months, 12 months and 36 months). Friedman and Wilcoxon non-parametric tests were used to compare the differences in the ratings of each restorative system at baseline and at 6, 12 and 36 months. The ‘Alpha’ error was set at *0.05*. Fisher Exact Test was used to confirm that the cavity classes and the tooth types were equitably distributed among groups *(-p-value >.05)*. The software program used was IBM SPSS 18 for Windows (IBM Corporation, Armonk, NY, USA)

## Results

A total of 138 restorations were initially placed in 46 patients. They were distributed evenly among the different types of teeth, 60 (43.5%) restorations were placed in premolars and 78 (56.5%) in molars (Tables 1 and 5). Ninety-one restorations were evaluated at the 36 months recall with a 66% recall rate. Overall, 15 patients did not attend the last recall for the following reasons: four relocated, one started an orthodontic treatment and 10 refrained from attending their appointments for unknown reasons (Figure 1). Seven restorations, three SBU/FZXT, three GB/GP and one OBX/HU failed during 36 months of clinical service. The reasons for failure were marginal fracture and secondary caries as shown in Table 6. Success rates were: 93.5% (SBU/FZXT), 96.6% (GB/GP) and 96.8% (OBX/HU). The clinical evaluation ratings of the restorations are shown in Table 7. All restorations obtained Alpha ratings, except for 14 restorations that rated Bravo at baseline (marginal adaptation: 3, anatomical form: 2, surface texture: 2 and colour match: 7). Failed restorations were replaced, but included in the statistical analysis as ‘Delta’. In general, deterioration in all parameters was observed for SBU/FZXT restorations, except for the colour match criterion where no significant change was recorded. While in groups GB/GP and OBX/HU, all parameters did not show any significant change except for colour match for GB/GP restorations. A significant difference between the three groups was registered at 6, 12 and 36 months, for marginal adaptation and surface texture and at 36 months for marginal discoloration (Table 5).

**Table 5.** Descriptive statistics of the evaluated parameters for each restorative system.

	SBU/FZXT	GB/GP	OBX/HU	*p Value*
Anatomical Form				
Evaluation period				
Baseline	1.02 ± 0.147^4^ (*n* = 46)	1.02 ± 0.147 (*n* = 46)	1.00 ± 0.000 (*n* = 46)	.604
6 months	1.07 ± 0.327^4,5^ (*n* = 46)	1.02 ± 0.147 (*n* = 46)	1.00 ± 0.000 (*n* = 46)	.360
12 months	1.11 ± 0.379^5,6^ (*n* = 46)	1.07 ± 0.327 (*n* = 46)	1.00 ± 0.000 (*n* = 46)	.127
36 months	1.15 ± 0.470^6^ (*n* = 31)	1.04 ± 0.206 (*n* = 29)	1.04 ± 0.295 (*n* = 31)	.182
*-p-value*	*0.036*	*0.801*	*0.392*	
Marginal Adaptation				
Baseline	1.04 ± 0.206^4^ (*n* = 46)	1.02 ± 0.147 (*n* = 46)	1.00 ± 0.000 (*n* = 46)	.362
6 months	1.17 ± 0.437^2/4,5^ (*n* = 46)	1.11 ± 0.482^1,2^ (*n* = 46)	1.00 ± 0.000^1^ (*n* = 46)	.020
12 months	1.30 ± 0.511^3/5,6^ (*n* = 46)	1.17 ± 0.570^2^ (*n* = 46)	1.02 ± 0.147^1^ (*n* = 46)	.001
36 months	1.48 ± 0.752^2/6^ (*n* = 31)	1.09 ± 0.354^1^ (*n* = 29)	1.11 ± 0.482^1^ (*n* = 31)	<.001
*-p-value*	*<0.001*	*0.156*	*0.066*	
Colour Match				
Baseline	1.04 ± 0.206 (*n* = 46)	1.02 ± 0.147^4^ (*n* = 46)	1.09 ± 0.285 (*n* = 46)	.351
6 months	1.04 ± 0.206 (*n* = 46)	1.02 ± 0.147^4^ (*n* = 46)	1.09 ± 0.285 (*n* = 46)	.351
12 months	1.07 ± 0.250 (*n* = 46)	1.04 ± 0.206^4^ (*n* = 46)	1.09 ± 0.285 (n = 46)	.702
36 months	1.11 ± 0.315 (*n* = 31)	1.17 ± 0.437^5^ (*n* = 29)	1.09 ± 0.285 (*n* = 31)	0.592
*-p-value*	*0.066*	*0.012*	–	
Marginal discoloration				
Baseline	1.00 ± 0.000^4^ (*n* = 46)	1.00 ± 0.000 (*n* = 46)	1.00 ± 0.000 (*n* = 46)	1.000
6 months	1.04 ± 0.206^4^ (*n* = 46)	1.02 ± 0.147 (*n* = 46)	1.00 ± 0.000 (*n* = 46)	.362
12 months	1.13 ± 0.341^4,5^ (*n* = 46)	1.07 ± 0.250 (*n* = 46)	1.04 ± 0.206 (*n* = 46)	.279
36 months	1.26 ± 0.444 ^2/5^ (*n* = 31)	1.07 ± 0.250^1^(*n* = 29)	1.04 ± 0.206^1^ (*n* = 31)	.002
*-p-value*	*<0.001*	*0.234*	*0.112*	
Surface Texture				
Baseline	1.04 ± 0.206^4^ (*n* = 46)	1.00 ± 0.000 (*n* = 46)	1.00 ± 0.000 (*n* = 46)	.133
6 months	1.13 ± 0.341^2/^^5^ (*n* = 46)	1.04 ± 0.206^1^ (*n* = 46)	1.00 ± 0.000^1^ (*n* = 46)	.025
12 months	1.13 ± 0.341^2/5^ (*n* = 46)	1.07 ± 0.250^1,2^ (*n* = 46)	1.00 ± 0.000^1^ (*n* = 46)	.041
36 months	1.13 ± 0.341^2/5^ (*n* = 31)	1.11 ± 0.315^2^ (*n* = 29)	1.00 ± 0.000^1^ (*n* = 31)	.048
*-p-value*	*0.007*	*0.112*	–	
Secondary caries				
Baseline	1.00 ± 0.000 (*n* = 46)	1.00 ± 0.000 (*n* = 46)	1.00 ± 0.000 (*n* = 46)	1.000
6 months	1.00 ± 0.000 (*n* = 46)	1.02 ± 0.147 (*n* = 46)	1.00 ± 0.000 (*n* = 46)	.368
12 months	1.02 ± 0.147 (*n* = 46)	1.04 ± 0.206 (*n* = 46)	1.00 ± 0.000 (*n* = 46)	.362
36 months	1.04 ± 0.206 (*n* = 31)	1.07 ± 0.250 (*n* = 29)	1.00 ± 0.000 (*n* = 31)	.236
*-p-value*	0.194	0.172	1.000	

1,2,3: Significant difference between the restorative materials (*p* < .05).

4,5,6: Significant difference in comparison with baseline for each restorative material (*p* < .05).

*n*: number of restorations.

**Table 6.** Failed restorations during the 36 months evaluation, tooth type, year, and reason for failure.

Restorative system	Cavity class/ Tooth	Number of failed restorations	Month(M)	Reasons
Scotchbond Universal	Class II M	1	12 M	Secondary caries
Filtek Z350 XT	Class II M	1	36 M	Marginal fracture
	Class II Pm	1	36 M	Marginal fracture
G-aenial bond	Class II M	1	6 M	Secondary caries
G-aenial Posterior	Class II M	1	12 M	Marginal fracture
	Class II M	1	12 M	Secondary caries
OptiBond XTR	Class II M	1	36 M	Marginal fracture
Herculite Ultra				

M: Molar; Pm: Premolars.

**Table 7.** Number and percentages of restorations for each group evaluated according to the modified USPHS criteria.

SBU/FZXT					GB/GP				OBX/HU			
	BL	6 months	12 months	36 months	BL	6 months	12 months	36 months	BL	6 months	12 months	36 months
Anatomical form												
A	45 (97.8%)	44 (95.7%)	42 (91.3%)	28 (90.3%)	45 (97.8%)	45 (97.8%)	44 (95.7%)	29 (100.0%)	46 (100%)	46 (100%)	46 (100%)	30 (96.8%)
B	1 (2.2%)	1 (2.2%)	3 (6.5%)	1 (2.2%)	1 (6.5%)	1 (2.2%)	1 (2.2%)	0 (0%)	0 (0%)	0 (0%)	0 (0%)	0 (0%)
C	0 (0%)	1 (2.2%)	1 (2.2%)	2 (4.3%)	0 (0%)	0 (0%)	1 (2.2%)	0 (0 %)	0 (0%)	0 (0%)	0 (0%)	1 (3.2%)
Marginal adaptation												
A	44 (95.7%)	39 (84.8%)	33 (71.7%)	18 (58.1%)	45 (97.8%)	43 (93.5%)	43 (93.5%)	27 (93.1%)	46 (100%)	46 (100%)	45 (97.8%)	29 (93.5%)
B	2 (4.3%)	6 (13.0%)	12 (26.1%)	11 (35.5%)	1 (2.2%)	2 (4.3%)	1 (2.2%)	1 (3.4%)	0 (0%)	0 (0%)	1 (2.2%)	1 (3.2%)
C	0 (0%)	1 (2.2%)	1 (2.2%)	0 (0%)	0 (0%)	1 (2.2%)	1 (2.2%)	1 (3.4%)	0 (0%)	0 (0%)	0 (0%)	0 (0%)
D	0 (0%)	0 (0%)	0 (0%)	2 (6.5%)	0 (0%)	0 (0%)	1 (2.2%)	0 (0%)	0 (0%)	0 (0%)	0 (0%)	1 (3.2%)
Colour match												
A	44 (95.7%)	44 (95.7%)	43 (93.5%)	27 (87.1%)	45 (97.8%)	45 (97.8%)	44 (95.7%)	26 (89.7%)	42 (91.3%)	42 (91.3%)	42 (91.3%)	28 (90.3%)
B	2 (4.3%)	2 (4.3%)	3 (6.5%)	4 (12.9%)	1 (2.2%)	1 (2.2%)	2 (4.3%)	2 (6.9%)	4 (8.7%)	4 (8.7%)	4 (8.7%)	3 (9.7%)
C	0 (0%)	0 (0%)	0 (0%)	0 (0%)	0 (0%)	0 (0%)	0 (0%)	1 (3.4%)	0 (0%)	0 (0%)	0 (0%)	0 (0%)
Marginal discoloration												
A	46 (100%)	44 (95.7%)	40 (87.0%)	22 (71.0%)	46 (100%)	45 (97.8%)	43 (93.5%)	26 (89.7%)	46 (100%)	46 (100%)	44 (95.7%)	30 (96.8%)
B	0 (0%)	2 (4.3%)	6 (13.0%)	9 (29.0%)	0 (0%)	1 (2.2%)	3 (6.5%)	3 (10.3%)	0 (0%)	0 (0%)	2 (4.3%)	1 (3.2%)
Surface texture												
A	44 (95.7%)	40 (87.0%)	40 (87.0%)	27 (87.1%)	46 (100%)	44 (95.7%)	43 (93.5%)	26 (89.7%)	46 (100%)	46 (100%)	46 (100%)	31 (100.0%)
B	2 (4.3%)	6 (13.0%)	6 (13.0%)	4 (12.9%)	0 (0%)	2 (4.3%)	3 (6.5%)	3 (10.3%)	0 (0%)	0 (0%)	0 (0%)	0 (0%)
Secondary caries												
A	46 (100%)	46 (100%)	45 (97.8%)	30 (96.8%)	46 (100%)	45 (97.8%)	44 (95.7%)	28 (96.6%)	46 (100%)	46 (100%)	46 (100%)	31 (100.0%)
D	0 (0%)	0 (0%)	1 (2.2%)	1 (3.2%)	0 (0%)	1 (2.2%)	2 (4.3%)	1 (2.2%)	0 (0%)	0 (0%)	0 (0%)	0 (0%)

A: Alpha, B: Bravo, C: Charlie, D: Delta, SBU/FZXT: Scotch Bond Universal/Filtek Z350 XT, GB/GP: G- aenial Bond/ G-aenial Posterior, OBX/HU: Opti Bond XTR/Herculite Ultra.

## Anatomical form

In terms of the anatomical form criteria, there was a statistically significant decrease in ratings, during the 36 months, in Filtek Z350 XT composite restorations (SBU/FZXT group). Unlike the G-aenial Posterior (GB/GP group) and Herculite Ultra composite restorations (OBX/HU group) that showed an insignificant decrease during the evaluation periods. Four restorations (2 Filtek Z350 XT at 6 and 12 months, 1 G-aenial Posterior at 12 months and 1 Herculite Ultra at 36 months) with wear were detected and rated Charlie. However, statistically significant differences were not found between the three different composites from baseline to 36 months (*p >* .05). The restorations that maintained Alpha ratings were above 90% for all the restorative systems, in terms of anatomical form.

## Marginal adaptation

For marginal adaptation criteria, 81.3% of the composite restorations were rated Alpha after 36 months (SBU/FZXT =58.1%; GB/GP =93.1%; OBX/HU = 93.5%). However, a significant change was observed for the SBU/FZXT group and two restorations failed at 36 months (*p* < .05). Unlike the GB/GP and OBX/HU groups, their marginal adaptation variations were not significant after 36 months of clinical service *(p* > .05), though two restorations, 1 GB/GP and 1 OBX/HU, with marginal fracture were seen at 12 and 36 months respectively. A statistically significant difference between the groups (*p* < 0.05) was observed, from 6 to 36 months.

## Colour match

In terms of colour match, no change was recorded for any restoration from BL to 6 months. In the subsequent follow-ups, at 12 and 36 months, colour change was observed in Filtek Z350 XT and G-aenial Posterior composite restorations (SBU/FZXT and GB/GP groups) and was significant for only the G-aenial Posterior composite as 1 restoration had an obvious colour mismatch (*p* < .05). The Herculite Ultra restorations had no changes in colour, therefore, maintained their ratings after 36 months. In an intra group comparison, the difference between the three groups was not statistically significant at any of the recalls (*p* > .05) and restorations maintaining their alpha ratings were 87.1% for SBU/FZXT, 89.7% GB/GP and 90.3% OBX/HU groups.

## Marginal discoloration

Marginal discoloration was seen in 14 restorations and distributed as follows; 29% SBU/FZXT, 10.3% GB/GP and 3.2% OBX/HU. Superficial staining appeared first at 6 months. The numbers of restorations with stains increased significantly for the group SBU/FZXT only (*p* < .05). In the GB/GP group, staining was registered at 6 and 12 months but then remained the same till 36 months. In the OBX/HU group marginal staining was first seen after 12 months, followed by a minor decrease in number of restorations showing marginal discoloration after 36 months due to patient loss. The restorations in groups GB/GP and OBX/HU performed similarly but there was a significant difference between them and the SBU/FZXT group at the 36 months evaluation (*p* < .05).

## Surface texture

During the 36 months evaluation period, the composite restorations in the OBX/HU group, maintained an excellent surface texture (100%), unlike the SBU/FZXT and GB/GP restorations. In the SBU/FZXT group, a change in surface texture was observed and was statistically significant (*p* < 0.05) and 87.1% of the restorations maintained a good surface texture. A slight decrease was observed for the GB/GP composite restorations (89.3%), at the 6 and 12 months recalls but was statistically insignificant (*p* > .05). Furthermore, there was a statistically significant difference between the three groups at all the recalls (*p* < .05).

## Secondary caries

During the 36 months, there was no sign of secondary caries in any of the restored teeth in the OBX/HU group and 100% of the restorations were rated Alpha. Recurrent caries was detected as early as 6 months in one (2.2%) GB/GP restoration followed by another one (4.3%) at 12 months and none were detected at 36 months. In the SBU/FZXT group, recurrent caries was detected in one restoration (2.2%) only at 12 months recall. In general, there was no statistically significant difference between the three groups from baseline to 36 months (*p >* .05) and caries free restorations, rated Alpha were more than 96%.

## Discussion

Randomized clinical trials are fundamental in assessing the clinical performance of restorative materials. These materials are exposed to the variable conditions of the oral cavity. Therefore, clinical trials are preferred over laboratory tests. This study provides significant scientific data on the behaviour of different resin composites bonded with self-etch adhesives. They were all applied in SE mode. Considering the results obtained, the null hypothesis was rejected, as significant differences in performance were observed between the three restorative systems.

The poorest clinical outcome was seen for the marginal adaptation parameter, and SBU/FZXT restorations presented the most significant marginal deteriorations, where only 58.1% of their restorations maintained Alpha ratings after 36 months. Failure due to marginal fractures were seen in four (2.9%) restorations. They occurred in class II restorations at the cavity margins. The first marginal fracture was seen at 6 months, in one GB/GP restoration (Figure 4). Subsequently, two SBU/FZXT and one OBX/HU marginal fractures occurred after 36 months. Restorations that failed earlier in the study were not registered, as the patients did not attend the final recall (Table 7). Individuals enrolled in this study did not have any parafunctional habits according to the selection criteria. All of the restorations were in contact with natural opposing dentition, but most of the occlusal contacts were located at the restoration margins, which could have increased the fracture incidence. Moreover, overfilling the cavity and inadequately assessing the occlusion increased stress concentration which resulted in premature failure [19].

**Figure 2.**
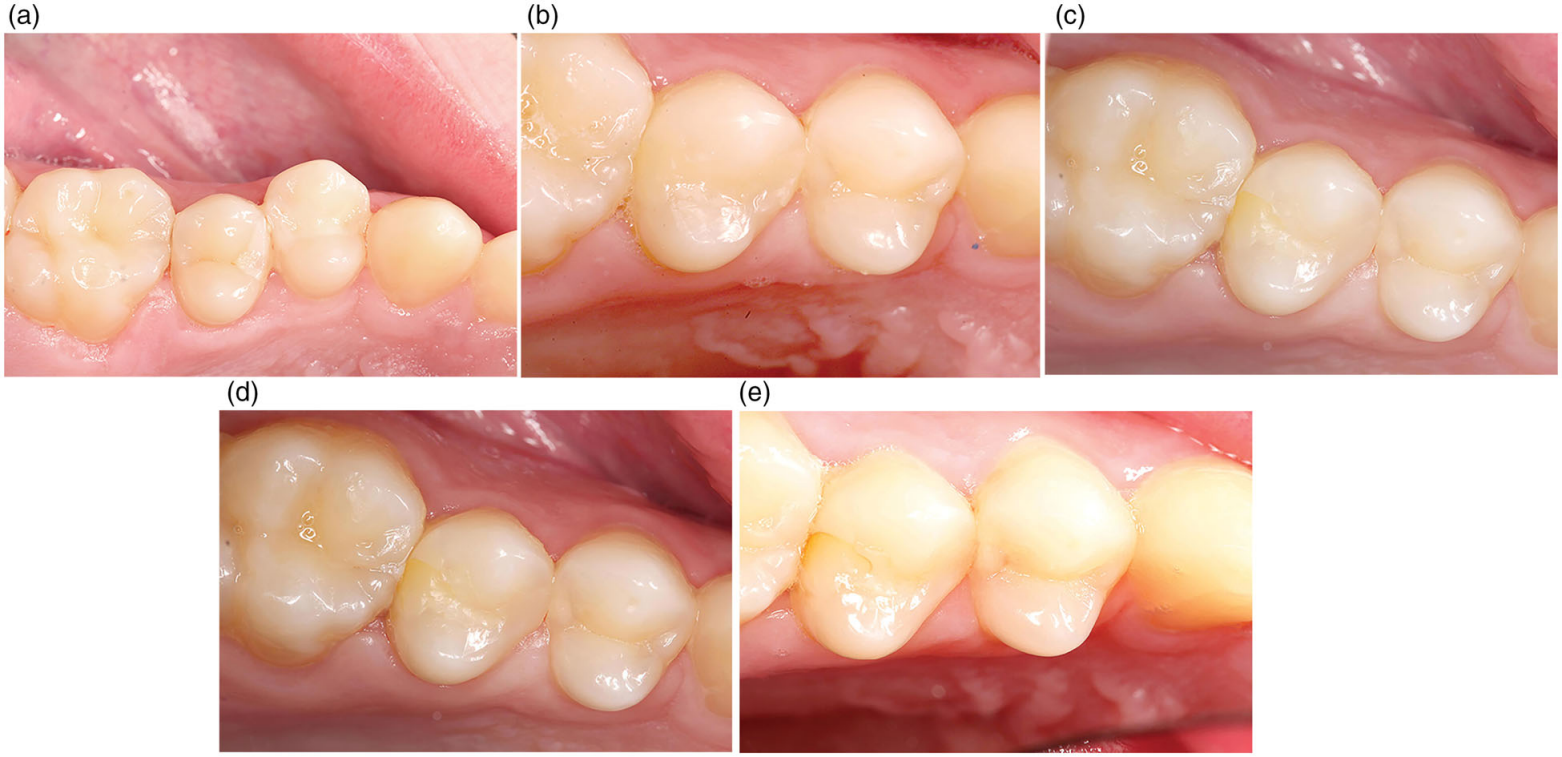
(a) Tooth 15 MOD (SBU/FZXT) preoperative. (b) Tooth 15 MOD (SBU/FZXT) at Baseline. (c) Tooth 15 MOD (SBU/FZXT) at 6 months. (d) Tooth 15 MOD (SBU/FZXT) at 12 months. (e) Tooth 15 MOD (SBU/FZXT) at 36 months.

**Figure 3.**
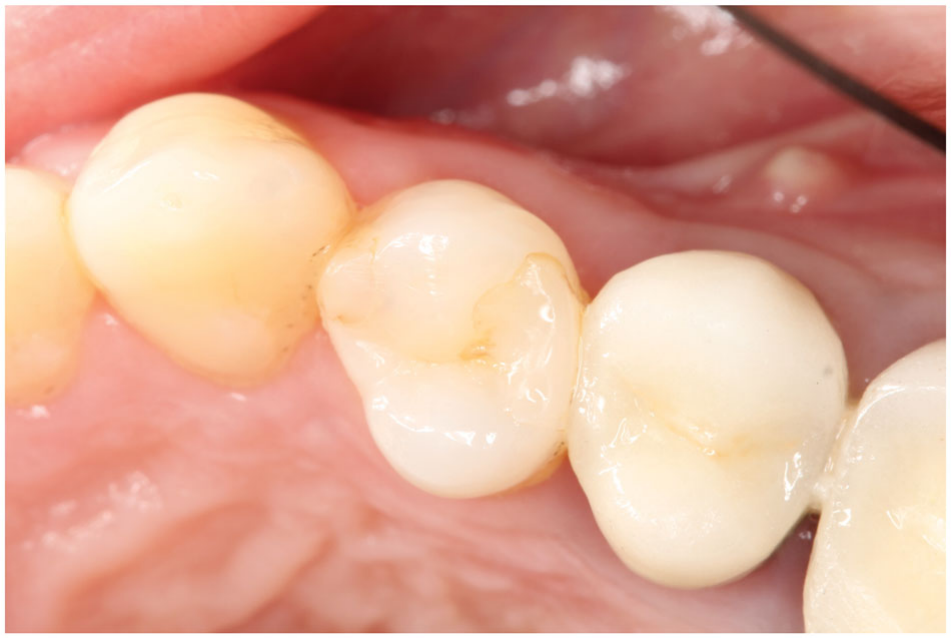
Tooth 14 OD (SBU/FZXT) superficial marginal discoloration at 36 months.

Marginal discolorations were seen in the three groups (SBU/FZXT, GB/GP and OBX/HU). The staining was superficial and located both occlusal and proximal at the buccal and lingual wall perimeter of Class II restorations, as shown in Figures 2 and 3. Marginal discoloration was more frequently observed in the SBU/FZXT composite restorations. In general, marginal discrepancies occurred more often in restorations bonded with self-etch adhesives. They form weak enamel bonds that promote debonding at restoration margins. This facilitated the entry of contaminants, resulting in marginal discolorations, which can explain our findings [7,9,10,14,20–22]. Ruschel et al., reported that the clinical performance of universal adhesives was independent of the application mode [23]. Conversely, in another study, poor marginal adaptations and increased marginal discolorations were observed only when the universal adhesives were applied in SE mode [24]. Variations can be encountered between materials in the same class of adhesives as their behaviour is product-dependent [25]. Studies confirmed that, selective enamel etching with phosphoric acid improved the effectiveness of mild SE and universal adhesives [13,26]. It must be noted that, when the present study was designed and performed the selective enamel etching technique was not yet recommended as a potential solution to improve the enamel bond. Therefore, the adhesives in the restorative systems tested were all applied in SE mode. According to the manufacturer, the pH of Scotchbond Universal is 2.7 and that of Optibond XTR and of G-aenial Bond are 2.4 and 1.5 respectively. Thus, when SE mode was used, restorations bonded with G-aenial Bond were expected to have a superior marginal adaptation compared to the universal adhesive and the two-step self-etch adhesive. The lower the pH of the adhesive, the more efficient is the selective enamel etching [7]. Conversely, in this study, the OBX/HU group with the OptiBond XTR adhesive performed better for all evaluated parameters, especially for the marginal adaptation. These results can be partly explained by the use of this particular two-step self-etch adhesive. OptiBond XTR consists of a separate hydrophobic component and contains a GPDM monomer, which can etch the enamel efficiently. For these reasons, OptiBond XTR achieved comparable bond strength to E&R adhesives, which adds to their overall success [27]. Unlike, the OptiBond XTR adhesive, Scotchbond Universal and G-aenial Bond adhesive systems have all their components combined. This creates instability and increases solubility, thus, reducing their mechanical properties [28–31]. In line with our results, a recent systemic review and meta-analysis reported that the one-step adhesive system exhibited lower bond strength than the two-step or three-step bonding systems after 1-year evaluation [32]. Another study by Perdigão et al., showed that one-step self-etch adhesives had significant inferior marginal integrity, colour match, surface texture, and occlusal function than the two-step etch-and-rinse adhesive systems [33]. Other studies with opposing results did not report any significant differences in the performance between the two-step SE OptiBond XTR adhesive and one-step SE All-Bond Universal adhesive in class II restorations [13]. The Scotchbond Universal adhesive contains 10-MDP (10-methacryloyloxydecyl dihydrogen phosphate) functional monomer, which imparts this adhesive its versatility [9,14,34]. It also contains HEMA (2-hydroxyethyl methacrylate) which facilitates the attraction of water from the dentinal tubules and the dissolution of the acidic monomers. Thus, their etching abilities are affected, resulting in a defective adhesion to enamel, which partly explains the poor performance of the Scotchbond universal adhesive [35]. Previous studies have reported no significant difference in the clinical behaviour of resin composites bonded with universal adhesives in any mode [36,37]. So far, mild SE adhesives and universal adhesives have proven to be clinically effective [7,38]. However, oversimplification of the adhesive systems and clinical procedures can compromise the clinical outcome. For this reason, more clinical studies are required to gain a better understanding on the performance of different marketed products.

**Figure 4.**
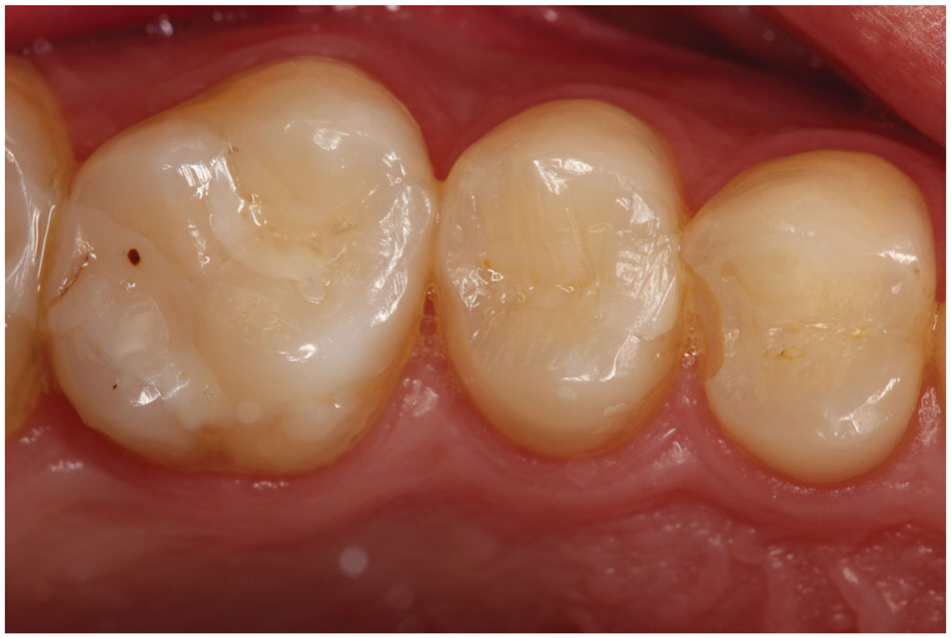
Tooth 24 OD (GB/GP) Marginal fracture at 6 months.

Besides the adhesive system used, other factors that affect restoration performance are composite-related. A major problem with resin composites is the polymerization shrinkage. This phenomenon, has significant implications on the long-term performance of restorations. which can lead to poor marginal adaptation, marginal discolorations, fractures, microleakage and recurrent caries, as seen in this study [39]. The composites used have comparable percentages of fillers by volume: Filtek Z350 XT (63.3%), G-aenial Posterior (65%) and Herculite Ultra (61%). Hence, similarity in properties and clinical behaviour was predicted [40]. Surface roughness was detected in some nanofilled Filtek Z350 XT resin composite restorations in the SBU/FZXT group and in the microhybrid G-aenial Posterior resin composite restorations in the GB/GP group but was statistically significant only for SBU/FZXT restorations. The finishing and polishing procedures are most likely the reason behind surface roughness, as two restorations were rated bravo at baseline. In previous studies, surface irregularity was frequently seen in Filtek Z350 XT restorations [41,42]. Additionally, the type of resin matrix, fillers and degree of conversion can influence the surface texture of the restorations [43]. A rough surface texture causes composite pigmentation from food intake, consequently colour mismatch with the adjacent teeth is seen in some restorations. While many clinical studies used NCCLs, the present study used class I and II lesions, that are stress-bearing posterior cavities and are frequently restored, for a better understanding of the restorative systems performance [36]. The C-factor is higher for class I cavities, yet failure occurred only in class II cavities and particularly at the gingival margins [44]. In this study, the gingival margins were mostly located in dentin with insufficient enamel, hence establishing a good bond strength at this region is important to avoid gap formation [44]. Therefore, the location of restoration margins can impact marginal integrity [45]. Besides marginal fracture, secondary caries was also a common reason of restoration failure [14]. It was detected as early as 6 months for a GB/GP restoration. Possible reasons could be that primary caries were not completely removed or that the patient presented bad oral hygiene, which may have increased the risk of recurrent caries [46].

Despite the strength of our study in using three different restorative systems which were evaluated clinically for 3 years, the current findings should be seen in the light of the following limitations: the modified USPHS criteria was employed as examiners were familiar with it and have used it before. However, its insensitivity when compared to the FDI evaluation criteria may have given a false impression of good performance in some cases leading to inaccurate results. Further follow-ups are required, as longer clinical trials can distinguish the restoration flaws more precisely, irrespective of the evaluation system used [15].

In conclusion, the three restorative systems have comparable clinical effectiveness and success rates. Except for marginal integrity where restorations bonded with the universal adhesive and one-step self-etch adhesive had inferior marginal adaptations than the restorations bonded with the two-step self-etch adhesive. When comparing the parameters directly influenced by the type of composite used, such as anatomical form, colour match and surface texture, results were similar for the nanofilled, nanohybrid and microhybrid resin composites.

## References

[1] Bayne SC, Ferracane JL, Marshall GA-O, et al. The evolution of dental materials over the past century: silver and gold to tooth color and beyond. J Dent Res. 2019;98(3):257–265. 30784370 10.1177/0022034518822808

[2] Tuncer S, Demirci M, Öztaş E, et al. Microhybrid versus nanofill composite in combination with a three step etch and rinse adhesive in occlusal cavities: five year results. Restor Dent Endod. 2017;42(4):253–263. 29142873 10.5395/rde.2017.42.4.253 PMC5682141

[3] Alzraikat H, Burrow MF, Maghaireh GA, et al. Nanofilled Resin Composite Properties and Clinical Performance: A Review. Oper Dent. 2018;43(4):E173–E190. 29570020 10.2341/17-208-T

[4] Demarco FF, Corrêa MB, Cenci MS, et al. Longevity of posterior composite restorations: Not only a matter of materials. Dent Mater. 2012;28(1):87–101. 22192253 10.1016/j.dental.2011.09.003

[5] Maran BM, de Geus JL, Gutiérrez MF, et al. Nanofilled/nanohybrid and hybrid resin-based composite in patients with direct restorations in posterior teeth: A systematic review and meta-analysis. J Dent. 2020;99:103407. 32526348 10.1016/j.jdent.2020.103407

[6] Çelik Ç, Arhun N, Yamanel K. Clinical evaluation of resin-based composites in posterior restorations: a 3-year study. Med Princ Pract. 2014;23(5):453–459. 25115230 10.1159/000364874 PMC5586919

[7] Van Meerbeek B, Yoshihara K, Van Landuyt K, et al. From Buonocore's pioneering acid-etch technique to self-adhering restoratives. a status perspective of rapidly advancing dental adhesive technology. J Adhes Dent. 2020;22(1):7–34. 32030373 10.3290/j.jad.a43994

[8] Perdigão J, Araujo E, Ramos R, et al. Adhesive dentistry: Current concepts and clinical considerations. J Esthet Restor Dent. 2021;33(1):51–68. 33264490 10.1111/jerd.12692

[9] Perdigão J, Ceballos L, Giráldez I, et al. Effect of a hydrophobic bonding resin on the 36-month performance of a universal adhesive-a randomized clinical trial. Clin Oral Invest. 2020;24(2):765–776. 10.1007/s00784-019-02940-x 31147827

[10] Loguercio AD, de Paula EA, Hass V, et al. A new universal simplified adhesive: 36-Month randomized double-blind clinical trial. J Dent. 2015; 43(9):1083–1092. 26159382 10.1016/j.jdent.2015.07.005

[11] Van Meerbeek B, Yoshihara K, Yoshida Y, et al. State of the art of self-etch adhesives. Dent Mater. 2011; 27(1):17–28. 21109301 10.1016/j.dental.2010.10.023

[12] Lührs AK, Guhr S, Borchers L, et al. Shear bond strength of self-etch adhesives to enamel with additional phosphoric acid etching. Oper Dent. 2008;33(2):155–162. 18435189 10.2341/07-63

[13] Van Dijken JW, Pallesen U. Three-year randomized clinical study of a one-step universal adhesive and a two-step self-etch adhesive in class ii composite restorations. J Adhes Dent. 2017;19(4):287–294. 28849796 10.3290/j.jad.a38867

[14] Cuevas-Suárez C, Da Rosa W, Lund R, et al. Bonding performance of universal adhesives: an updated systematic review and meta-analysis. J Adhes Dent. 2019;21(1):7–26. 30799468 10.3290/j.jad.a41975

[15] de Paris Matos T, Perdigão J, de Paula E, et al. Five-year clinical evaluation of a universal adhesive: A randomized double-blind trial. Dent Mater. 2020;36(11):1474–1485. 32933775 10.1016/j.dental.2020.08.007

[16] Carlson RV, Boyd K, Webb DJ. The revision of the Declaration of Helsinki: past, present and future. Br J Clin Pharmacol. 2004;57(6):695–713. 15151515 10.1111/j.1365-2125.2004.02103.x PMC1884510

[17] Posterior composite resins. Council on dental materials, instruments, and equipment. J Am Dent Assoc. 1986;112(5):707–709. 3458787

[18] Ryge G, Snyder M. Evaluating the clinical quality of restorations. J Am Dent Assoc. 1973;87(2):369–377. 4515696 10.14219/jada.archive.1973.0421

[19] Bicalho AA, Tantbirojn D, Versluis A, et al. Effect of occlusal loading and mechanical properties of resin composite on stress generated in posterior restorations. Am J Dent. 2014;27(3):129–133. 25208359

[20] Akimoto N, Takamizu M, Momoi Y. 10-year clinical evaluation of a self-etching adhesive system. Oper Dent. 2007;32(1):3–10. 17288322 10.2341/06-46

[21] Schroeder M, Correa IC, Bauer J, et al. Influence of adhesive strategy on clinical parameters in cervical restorations: A systematic review and meta-analysis. J Dent. 2017;62:36–53. 28495559 10.1016/j.jdent.2017.05.006

[22] Peumans M, De Munck J, Van Landuyt K, et al. Thirteen-year randomized controlled clinical trial of a two-step self-etch adhesive in non-carious cervical lesions. Dent Mater. 2015;31(3):308–314. 25637318 10.1016/j.dental.2015.01.005

[23] Ruschel V, Shibata S, Stolf S, et al. Eighteen-month clinical study of universal adhesives in noncarious cervical lesions. Oper Dent. 2018;43(3):241–249. 29676975 10.2341/16-320-C

[24] Perdigão J, Kose C, Mena-Serrano AP, et al. A new universal simplified adhesive: 18-month clinical evaluation. Oper Dent. 2014;39(2):113–127. 23802645 10.2341/13-045-C

[25] Hickel R, Roulet J, Bayne S, et al. Recommendations for conducting controlled clinical studies of dental restorative materials. Science Committee Project 2/98–FDI World Dental Federation Study Design (Part I) and Criteria for Evaluation (Part II) of Direct and Indirect Restorations Including Onlays and Partial Crowns. 2007;9(6):546. 18341239

[26] Jacker-Guhr S, Sander J, Luehrs AK. How "Universal" is adhesion? Shear bond strength of multi-mode adhesives to enamel and dentin. J Adhes Dent. 2019;21(1):87–95. 30799475 10.3290/j.jad.a41974

[27] Hoshika S, Kameyama A, Suyama Y, et al. GPDM- and 10-MDP-based self-etch adhesives bonded to bur-cut and uncut enamel immediate and aged µTBS. J Adhes Dent. 2018;20(2):113–120. 29675517 10.3290/j.jad.a40307

[28] Yoshikawa T, Sadr A, Tagami J. Effects of C-factor on bond strength to floor and wall dentin. Dent Mater J. 2016;35(6):918–922. 27725368 10.4012/dmj.2016-111

[29] Mine A, De Munck J, Cardoso M, et al. Bonding effectiveness of two contemporary self-etch adhesives to enamel and dentin. J Dent. 2009; 37(11):872–883. 19683377 10.1016/j.jdent.2009.06.020

[30] Vanajasan P, Dhakshinamoorthy M, Subba Rao C. Factors affecting the bond strength of self-etch adhesives: A meta-analysis of literature. J Conserv Dent. 2011;14(1):62–67. 21691509 10.4103/0972-0707.80746 PMC3099118

[31] Giannini M, Makishi P, Ayres AP, et al. Self-etch adhesive systems: a literature review. Braz Dent J. 2015;26(1):3–10. 25672377 10.1590/0103-6440201302442

[32] Masarwa N, Mohamed A, Abou-Rabii I, et al. Longevity of self-etch dentin bonding adhesives compared to etch-and-rinse dentin bonding adhesives: a systematic review. J Evid Based Dent Pract. 2016;16(2):96–106. 27449836 10.1016/j.jebdp.2016.03.003

[33] Perdigão J, Dutra-Corrêa M, Anauate-Netto C, et al. Two-year clinical evaluation of self-etching adhesives in posterior restorations. J Adhes Dent. 2009;11(2):149–159. 19492717

[34] Alex G. Universal adhesives: the next evolution in adhesive dentistry? Compend Contin Educ Dent. 2015;36(1):15–26. 25822403

[35] Manarte-Monteiro P, Domingues J, Teixeira L, et al. Multi-mode adhesives performance and success/retention rates in NCCLs restorations: randomised clinical trial one-year report. Biomater Investig Dent. 2019;6(1):43–53. 10.1080/26415275.2019.1684199 PMC6964776 31998871

[36] Carvalho AA, Leite MM, Zago JKM, et al. Influence of different application protocols of universal adhesive system on the clinical behavior of Class I and II restorations of composite resin - a randomized and double-blind controlled clinical trial. BMC Oral Health. 2019;19(1):252. 31752813 10.1186/s12903-019-0913-3 PMC6868695

[37] Yamauchi K, Tsujimoto A, Jurado CA, et al. Etch-and-rinse vs self-etch mode for dentin bonding effectiveness of universal adhesives. J Oral Sci. 2019;61(4):549–553. 31631096 10.2334/josnusd.18-0433

[38] Delbons FB, Perdigão J, Araujo E, et al. Randomized clinical trial of four adhesion strategies in posterior restorations-18-month results. J Esthet Restor Dent. 2015;27(2):107–117. 25627581 10.1111/jerd.12135

[39] Balkaya H, Arslan S. A two-year clinical comparison of three different restorative materials in class II cavities. Oper Dent. 2019;45(1):1559–2863. 10.2341/19-078-C 31738696

[40] Ferracane JL. Resin composite-state of the art. Dent Mater. 2011;27(1):29–38. 21093034 10.1016/j.dental.2010.10.020

[41] Patel B, Chhabra N, Jain D. Effect of different polishing systems on the surface roughness of nano-hybrid composites. J Conserv Dent. 2016;19(1):37–40. 26957791 10.4103/0972-0707.173192 PMC4760010

[42] Hoseinifar R, Mortazavi-Lahijani E, Mollahassani H, et al. One year clinical evaluation of a low shrinkage composite compared with a packable composite resin: a randomized clinical trial. J Dent. 2017;14(2):84–91. PMC5662513 29104599

[43] Ehrmann E, Medioni E, Brulat-Bouchard N. Finishing and polishing effects of multiblade burs on the surface texture of 5 resin composites: microhardness and roughness testing. Restor Dent Endod. 2019;44(1):e1. 30834223 10.5395/rde.2019.44.e1 PMC6387893

[44] Bohaty BS, Ye Q, Misra A, et al. Posterior composite restoration update: focus on factors influencing form and function. Clin Cosmet Investig Dent. 2013;5:33–42. 10.2147/CCIDE.S42044 PMC3666491 23750102

[45] Hayashi M, Wilson NH. Marginal deterioration as a predictor of failure of a posterior composite. Eur J Oral Sci. 2003;111(2):155–162. 12648268 10.1034/j.1600-0722.2003.00020.x

[46] Nedeljkovic I, De Munck J, Vanloy A, et al. Secondary caries: prevalence, characteristics, and approach. Clin Oral Investig. 2020;24(2):683–691. 10.1007/s00784-019-02894-0 31123872

